# Cross-kingdom interaction between *Candida albicans* and oral bacteria

**DOI:** 10.3389/fmicb.2022.911623

**Published:** 2022-11-03

**Authors:** Qian Du, Biao Ren, Xuedong Zhou, Ling Zhang, Xin Xu

**Affiliations:** ^1^State Key Laboratory of Oral Diseases, National Clinical Research Center for Oral Diseases, West China Hospital of Stomatology, Sichuan University, Chengdu, China; ^2^Department of Cariology and Endodontics, West China Hospital of Stomatology, Sichuan University, Chengdu, China; ^3^Stomatology Hospital, School of Stomatology, Zhejiang University School of Medicine, Zhejiang Provincial Clinical Research Center for Oral Diseases, Key Laboratory of Oral Biomedical Research of Zhejiang Province, Cancer Center of Zhejiang University, Hangzhou, China

**Keywords:** *Candida albicans*, bacteria, co-infection, biofilm, oral diseases

## Abstract

*Candida albicans* is a symbiotic fungus that commonly colonizes on oral mucosal surfaces and mainly affects immuno-compromised individuals. Polymicrobial interactions between *C. albicans* and oral microbes influence the cellular and biochemical composition of the biofilm, contributing to change clinically relevant outcomes of biofilm-related oral diseases, such as pathogenesis, virulence, and drug-resistance. Notably, the symbiotic relationships between *C. albicans* and oral bacteria have been well-documented in dental caries, oral mucositis, endodontic and periodontal diseases, implant-related infections, and oral cancer. *C. albicans* interacts with co-existing oral bacteria through physical attachment, extracellular signals, and metabolic cross-feeding. This review discusses the bacterial–fungal interactions between *C. albicans* and different oral bacteria, with a particular focus on the underlying mechanism and its relevance to the development and clinical management of oral diseases.

## Introduction

The oral cavity is one of the main sites of microorganisms colonization on the human body. More than 700 species of microorganisms can be detected in the human oral cavity, including bacteria, fungi, viruses, mycoplasma, rickettsia, and protozoa (Dewhirst et al., 2010). Microbiota that resides in the oral cavity can be symbiotic, competitive, and antagonistic to maintain the balance of microecology, which determines oral health and the development of biofilm-related oral diseases. Oral microbiota can be opportunistic pathogens when the oral microenvironment or personal oral hygiene changes (Wolcott et al., 2013). Recently, cross-kingdom interactions between fungi and oral bacteria have drawn increasing attention. *Candida albicans* can interact with a variety of oral microbes and their interactions are interdependent and mutually beneficial rather than unidirectional. These polymicrobial interactions have been demonstrated in the pathogenesis of biofilm-related oral diseases, including dental caries, oral candidosis, endodontic diseases, periodontitis, implant-related infections, and oral cancer (Bamford et al., 2009; Shirtliff et al., 2009; Morales and Hogan, 2010; Peleg et al., 2010; Harriott and Noverr, 2011; Diaz et al., 2012; Koo et al., 2018; Lohse et al., 2018).

**FIGURE 1:**
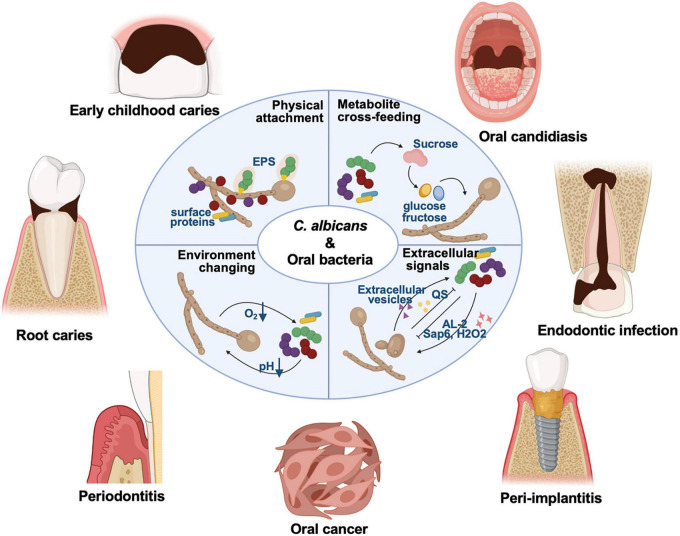
*C. albicans* can interact with a variety of oral microbes, and these cross-kingdom interactions have been demonstrated involved in the pathogenesis of oral diseases for years, including dental caries, oral candidosis, endodontic disease, periodontitis, and other biofilm-related oral diseases. *C. albicans* can interact with oral bacteria *via* physical attachment through fungal cell walls (e.g., surface proteins and EPS), extracellular signals, metabolite cross-feeding, and environment changing.

*C. albicans* is a symbiotic fungus commonly colonizing on the mucosal surfaces of living bodies. The detection rate of *C. albicans* in the healthy population is 18.5∼40.9% (Mun et al., 2016; Zomorodian et al., 2016; Babatzia et al., 2020). Individuals with compromised immune systems, such as HIV-positive individuals, newborns, and the elderly, are susceptible to *C. albicans* infection (Shay et al., 1997; Zomorodian et al., 2016; Diaz et al., 2017; Babatzia et al., 2020; Vila et al., 2020). *C. albicans* biofilms contain yeast, pseudo-hyphal, and hyphal form cells, surrounded by extracellular matrix (Wang, 2015; Lohse et al., 2018). After adherence to the surface, *C. albicans* cells proliferate in the form of yeast and begin to form hyphal, elongating and proliferating throughout the biofilm maturation process (Lohse et al., 2018). The yeast-to-hypha transition is widely recognized as a key virulence trait of *C. albicans* associated with biofilm formation (Garcia-Sanchez et al., 2004). The synergistic effects of *C. albicans* and commensal bacteria have been well-studied in the context of importance to the microbiological community, which impact on the virulence of polymicrobial biofilms and antibiotic resistance (Morales and Hogan, 2010; Diaz et al., 2014; Allison et al., 2016; Janus et al., 2016; Koo et al., 2018). *C. albicans* can interact with oral bacteria *via* physical attachment through fungal cell walls (e.g., surface proteins and extracellular polysaccharides, EPS), extracellular signals, metabolite cross-feeding, and environmental changes (Figure 1; Allison et al., 2016; Koo et al., 2018; Khan et al., 2021). Here, we discusses recent findings on the *C. albicans* mutualistic interactions with oral commensal bacteria, particularly focusing on the underlying mechanisms and relevance to biofilm-related oral diseases, aiming to provide new insights into prevention and treatment strategies for oral diseases.

## Key factors mediating cross-kingdom interactions between *C. albicans* and oral bacteria

### Surface proteins

**FIGURE 2:**
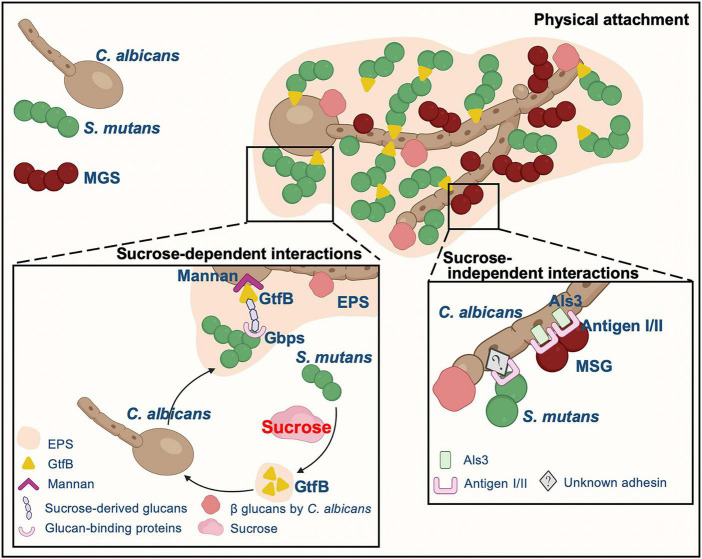
The typical physical attachment mechanisms underlying the interaction between *C. albicans* and streptococci. *C*. *albicans* physically interacts with MGS through proteins/receptors on cell wall surface of both fungi and bacteria whereas EPS is critical to the mutualistic interaction between *C. albicans* and *S. mutans* instead of cell-cell physical adhesion.

Cell-cell adhesion is one of the main and common factors mediating *C. albicans* and oral bacteria interactions, reciprocally aiding the colonization of both fungi and bacteria (Zijnge et al., 2010; Diaz et al., 2012; Falsetta et al., 2014; Xu et al., 2014a; Hwang et al., 2015; Nobbs and Jenkinson, 2015; Koo et al., 2018; Dige and Nyvad, 2019; Kim and Koo, 2020). Fungal cell walls are composed of different polysaccharides, including glucans, mannans, and chitin (Gow et al., 2017). There are a variety of adhesion proteins or receptors existing on the fungal cell wall. *C*. *albicans* physically interacts with oral microorganisms through these proteins or receptors to form a well-characterized structure, “cross-kingdom corncob” (Figure 2), such as mitis group streptococci (MGS, e.g., *Streptococcus gordonii*, *Streptococcus sanguinis*, and *Streptococcus oralis*), *Porphyromonas gingivalis*, and *Staphylococcus aureus* (Zijnge et al., 2010; Diaz et al., 2012; Peters et al., 2012; Falsetta et al., 2014; Xu et al., 2014a; Hwang et al., 2015; Nobbs and Jenkinson, 2015; Koo et al., 2018; Dige and Nyvad, 2019; Kim and Koo, 2020). Hyphae formation has been confirmed to be the preferred *C. albicans* morphotype of these microorganisms that adhere to (Brady et al., 2010; Silverman et al., 2010; Peters et al., 2012; Xu et al., 2014a; Bamford et al., 2015; Koo et al., 2018). Visualized by multi-color fluorescence microscopy, numerous clusters of either *S. oralis* or *S. gordonii* are observed forming around *C. albicans* hyphae within a few hours with a sufficient supply of nutrients (Bamford et al., 2009; Diaz et al., 2012), and even in poor nutrient microconditions, *S. oralis* can also bind to *Candida* germ tubes (Diaz et al., 2012). *C. albicans* hyphae-specific cell wall adhesins Als and Hwp1 appear to mediate the binding of this fungus to oral bacteria, such as *S. gordonii*, *S. oralis*, *P. gingivalis*, and *S. aureus*, and two antigen I/II family members of *Streptococcal* cell-surface adhesins, SspA and SspB, are illustrated to be the key points of interaction with *C*. *albicans* hyphae (Silverman et al., 2010; Peters et al., 2012; Xu et al., 2014a; Bamford et al., 2015; Xu et al., 2017; Koo et al., 2018; Bartnicka et al., 2019). Specifically, *S. gordonii* SspB protein directly interacts with *C. albicans* through the N-terminal domain of Als3 on the *C. albicans* hyphal filament surface (Silverman et al., 2010; Bamford et al., 2015). Meanwhile, *C. albicans* cell wall mannoproteins and O-mannosylation contribute to the development of inter-kingdom biofilm (Dutton et al., 2014). *C. albicans* O-mannosylation deficient strain (*mnt1*Δ *mnt2*Δ mutant) has defective functionality of adhesins, and its hyphal filaments do not interact with SspB adhesin on the *S. gordonii* surface or *C. albicans* Als3/Hwp1 protein, which is different from *C. albicans* wild-type hyphae (Dutton et al., 2014). Furthermore, the aspartyl proteinase Sap9, which is involved in designing of mono- and dual-species biofilms architecture (Dutton et al., 2016), is absent in this mutant. Increased gene expression of *ALS3* and *HWP1* has also been observed in *C. albicans* when co-cultured with *P. gingivalis* (Bartnicka et al., 2019), and the interaction of these two microorganisms occurs directly through the fungal adhesin Als3 and gingipain RgpA (Bartnicka et al., 2019). Unlike MGS, antigen I/II has been shown to mediate the dual-species biofilm interaction of *C. albicans* and *S. mutans* in an Als1/Als3-independent manner, and the underlying mechanism needs further exploration (Yang et al., 2018).

It is worth noting that although hyphae formation has been validated to be important in *C. albicans* mutualism effects with MGS (Brady et al., 2010; Silverman et al., 2010; Xu et al., 2014a; Bamford et al., 2015; Koo et al., 2018), hyphae formation may not affect the microorganism composition of more complicated polymicrobial communities (Xu et al., 2016; Montelongo-Jauregui et al., 2019; Du et al., 2021a). Efg1, a key *C. albicans* hyphae-associated morphological regulator, has been shown to be required for dual-species biofilm coaggregation between *C. albicans* and streptococci. Lacking hyphal formation, the *efg1*Δ/Δ strain forms significantly lighter biofilms with *S. oralis* compared with wild-type strain *in vitro. S. oralis* cannot upregulate *ALS1*, *ALS3*, and *HWP1* gene expression of *efg1*Δ/Δ strain (Xu et al., 2017). Whereas it has been observed that *S. oralis* is still able to promote *efg1*Δ/Δ strain colonization in oral mucosa of mice (Xu et al., 2016), and it is especially apparent in synthetic saliva culture conditions that *S. gordonii* and the *C. albicans efg1*Δ/Δ and *brg1*Δ/Δ filamentation-deficient mutants interact in a highly synergistic manner (Montelongo-Jauregui et al., 2019). The occurrence of this phenomenon suggests that hyphae formation is required for *Candida* interactions with bacteria *in vivo*, which may be influenced by of oral environment as well as the presence of commensal microbiota.

### Extracellular polysaccharides

Interestingly, different from MGS, synergetic collaboration between *C. albicans* and the main cariogenic bacteria *S. mutans* is dominated mainly by sucrose-dependent partnership (Figure 2). Sucrose significantly changes the adhesion pattern between *S. mutans* and *C. albicans*, increasing the connection between these two microorganisms. Sucrose allows *S*. *mutans* to produce EPS (also termed as water insoluble glucans), which is critical to the interaction between *C. albicans* and *S. mutans* rather than physical cell-cell adhesion (Bowen and Koo, 2011; Gregoire et al., 2011; Falsetta et al., 2014; Hwang et al., 2015; Hwang et al., 2017; Koo et al., 2018; Kim and Koo, 2020). RNA-Seq data have demonstrated that the presence of *C. albicans* in biofilms dramatically alters 393 gene expression in *S. mutans* and most of the upregulated genes are involved in carbohydrate transport and metabolic/catabolic processes (He et al., 2017). In the *C. albicans*/*S. mutans* mixed biofilm, *S. mutans* forms a large amount of microcolonies around fungal cells instead of directly adhering to the cell wall surface of *Candida*, and the microcolonies are enmeshed in an EPS-rich extracellular matrix (Falsetta et al., 2014; Hwang et al., 2017; Koo et al., 2018; Kim and Koo, 2020). In sucrose-limited environments, *C. albicans* adheres to the surface of *S. gordonii* about two times stronger than it adheres to *S. mutans*. However, in the presence of glucans, the binding force between *S. mutans* and *C. albicans* surfaces is dramatically elevated (∼6 folds) (Wan et al., 2021). *S*. *mutans* effectively synthesizes EPS from dietary sucrose through glucosyltransferases (Gtfs). All *S. mutans* Gtfs can adhere to *C. albicans* cell surfaces; among them, GtfB exhibits the greatest affinity (Bowen and Koo, 2011; Gregoire et al., 2011). The glucans produced abundantly by surface-bound GtfB on both organismal cells and tooth hard tissue concurrently enhance the efficiency and stability of *C. albicans* in attaching to and colonizing on the teeth. Simultaneously, the glucans on fungal cell walls in turn improve *S. mutans*-binding sites (Bowen and Koo, 2011; Gregoire et al., 2011; Falsetta et al., 2014; Huffines and Scoffield, 2020; Kim and Koo, 2020). *C. albicans* has a significantly larger surface area than bacteria with a plentiful source of Gtfs-binding sites for *S. mutans* colonization (Gregoire et al., 2011). GtfB adhesion to *C. albicans* is 2.5 folds stronger and 20 folds more stable than adhesion to *S. mutans* according to atomic force microscopy; however, there is an uneven distribution of GtfB-binding domains on the fungal surface (Hwang et al., 2015). On the other hand, *C. albicans* can also secrete polysaccharides that mediate mixed biofilm formation (Hall and Gow, 2013; Mitchell et al., 2015; Khoury et al., 2020). β-1,3-glucans synthesized by *Candida* also contribute to the co-cultured biofilm matrix structure (Falsetta et al., 2014; Khoury et al., 2020). *C. albicans* cell wall mannan provides sites for GtfB binding and function (Falsetta et al., 2014; Hwang et al., 2017; Kim et al., 2021). GtfB-binding domains have been demonstrated to reside in both the O- and N-terminal structures of mannans in *Candida* cell walls. *C*. *albicans pmt4*Δ/Δ and *och1*Δ/Δ strains exhibit functional defects in biological synthesis of both *N*- and *O*-linked mannans, and GtfB-binding properties are compromised compared to wild-type strain (Hwang et al., 2017). Meanwhile, mannan-degrading endo- and exo-enzymes reduce the GtfB-binding forces to *C. albicans* by about 15 folds, accompanied with thicker biofilm biomass, under the premise of eliminating the possibility of killing microorganisms (Kim et al., 2021).

### Extracellular signals

A range of extracellular signals appear to facilitate *C. albicans* synergistic interactions with oral bacteria, including signaling molecules, quorum sensing molecules (QS), and other factors (Table 1; Peleg et al., 2010; Harriott and Noverr, 2011; Xu et al., 2014a; Nobbs and Jenkinson, 2015; Lohse et al., 2018). QS is a microbial cell-to-cell communication process principally dependents on population density. As shown in an *in vitro* study, early in the exponential growth phase, about 4 h into culturing, *S. mutans* secretes competence-stimulating peptide (CSP) (Jarosz et al., 2009) and fatty acid signaling molecule trans-2-decenoic (SDSF) (Vilchez et al., 2010), which inhibit germ tube formation of *C. albicans*, thus favoring fungal proliferation. SDSF activity can be also detected in *S. mitis*, *S. oralis*, and *S. sanguinis* (Vilchez et al., 2010). In the later stage of biofilm maturity, the inhibitory germ tube formation effect on *C. albicans* disappears, and *C. albicans* produces a large number of hyphae and becomes more virulent (Jarosz et al., 2009; Vilchez et al., 2010). *S. mutans* can also upregulate *comC* of *C. albicans*, which encodes CSP (He et al., 2017) and the *S. gordonii comCDE* QS-system, which modulates the dual-species biofilm co-cultured with *C. albicans* (Jack et al., 2015). Meanwhile, when co-cultured with *S. gordonii*, *C. albicans* forms hyphae earlier and more extensively, which is mediated to a certain extent by autoinducer-2, a universal signaling molecule in interactions between bacterial species. *S. gordonii* effectively suppresses the inhibitory effect of farnesol on *C. albicans* filamentation in a LuxS-dependent pattern (Bamford et al., 2009). The role of QS molecule farnesol in *C. albicans* yeast-to-hyphae transition has long been recognized. A non-monotonic response to farnesol concentration is observed in *S. mutans* growth. In the *C. albicans/S. mutans* mixed biofilm, low levels of farnesol (∼25 μm) stimulate GtfB expression/activity and increase bacterial growth. In contrast, an abundance of farnesol (> 100 μm) inhibits *S. mutans* growth (Kim et al., 2017). Moreover, membrane vesicles produced by *S. mutans* can augment *C. albicans* biofilm development without significantly affect its planktonic growth (Wu et al., 2020).

**TABLE 1:** Summary of extracellular signals modulating *C. albicans* interactions between oral bacteria.

Molecule	Microorganism	Functional interaction with *C. albicans*	References
CSP	*S. mutans*	Inhibit hyphae and germ tube formation, keep *C. albicans* in yeast form, benefit to *C. albicans* proliferation	Jarosz et al., 2009
SDSF	*S. mutans*, MGS	Inhibit hyphae formation of *C. albicans* in early stage	Vilchez et al., 2010
farnesol	*C. albicans*	Low level: stimulate GtfB expression/activity and increase bacterial growth; High level: inhibit *S. mutans* growth	Kim et al., 2017
autoinducer-2	*S. gordonii*	Reduce the repressive effects of farnesol on hyphal formation of *C. albicans*	Bamford et al., 2009
membrane vesicles	*S. mutans*	Contribute to sucrose metabolism in *C. albicans*	Wu et al., 2020
hydrogen peroxide	*S. gordonii*	Oxidative and genotoxic stress; promote *C. albicans* filamentous growth	Bamford et al., 2009

### Metabolite cross-feeding and environmental change

*C. albicans* can use glucose as a carbon source but possesses insufficient ability to metabolize dietary sucrose. Streptococci may provide a nutritional source for *C. albicans* and enable its persistence under acidic condition (Bamford et al., 2009; Sztajer et al., 2014; He et al., 2017; Kim et al., 2017). *S. mutans* can rapidly breakdown sucrose to glucose and fructose, which can be utilized by *C. albicans*, thus elevating its growth (He et al., 2017; Kim et al., 2017). Sucrose and monosaccharides released by *S. mutans* from sucrose are depleted in spent medium of *S. mutans*/*C. albicans* dual-species biofilms at 10 h, whereas a large amount of carbohydrates remain in *S. mutans* single-species biofilms (Sztajer et al., 2014). With high salivary glucose levels, *Candida* colonization increases, insoluble EPS accumulates, and proteins and phospholipase activities increase (Brito et al., 2021). *S. mutans* and *C. albicans* dual-species biofilms produce lactate, along with small amounts of formate and fumarate as a result of carbohydrate metabolism, facilitating the growth of *S. mutans* and *C. albicans* within cariogenic biofilms, as both these organisms are acid-tolerant (He et al., 2017; Kim et al., 2017). Meanwhile, sucrose supplementation also reduces the inhibitory effect of sugar alcohols (Chan et al., 2020). In low-fermentable carbohydrate environments, *C. albicans* promotes the growth and biofilm formation of *S. gordonii* by elevating the enzymatic activities of cell wall-anchored glycoside hydrolases (GHs). GHs mediate the hydrolysis of glycoproteins, which is critical for the growth of *S. gordonii* in limited nutrient environment, such as saliva. Furthermore, *C. albicans* gene *TEC1* is critical for this cross-kingdom metabolic communication (Zhou et al., 2022).

At the same time, *C. albicans* consumes oxygen in the local environment to ensure strictly anaerobic conditions within the biofilm that favor the growth of anaerobes (Shirtliff et al., 2009; Fox et al., 2014; Janus et al., 2017; Bartnicka et al., 2019; Du et al., 2021a). Specifically, the presence of *C. albicans* alters the microbial composition of oral biofilms (Janus et al., 2017; Du et al., 2021a), increasing the abundance of strictly anaerobic *Veillonella*, *Prevotella*, *Leptotrichia*, and *Fusobacterium* genera under oxygen-rich conditions (Janus et al., 2017). The presence of *C. albicans* significantly increases *Streptococcus* in the saliva-derived biofilms in our previous study (Du et al., 2021a). *C. albicans* can also increase the viability of *P. gingivalis* biofilm by 20% in a normoxic environment (Bartnicka et al., 2019). Meanwhile, peptidoglycan fragments (Xu et al., 2008; Wang, 2013) and hydrogen peroxide (Nasution et al., 2008; Bamford et al., 2009) produced by catalase-negative bacteria can enhance *C. albicans* filamentation.

## Interactions between *C. albicans* and oral bacteria in oral diseases

### Dental caries

Dental caries, also known as tooth decay, is a representative biofilm-related oral disease and occurs as a result of microbial dysbiosis characterized with the enrichment of acidogenic pathogens and depletion of alkali-generating commensal microbes within the plaque biofilm (Pitts, 2016; Pitts et al., 2017; Bowen et al., 2018). Evidence has supported that *C. albicans* is closely related to the occurrence of dental caries. A recent meta-analysis reveals that the prevalence of dental caries in individuals carrying *Candida* is higher than those free of *Candida* in the oral cavity. Associations between oral *Candida* carriage and the occurrence of dental caries in children/adolescents and adults have been demonstrated (Eidt et al., 2020). A study of 132 patients with caries and 58 non-caries controls has demonstrated that patients with caries have greater *Candida* colonization than individuals without caries, and a significant clinical correlation between oral *Candida* carriage and dental caries incidence has been noted (De-la-Torre et al., 2016). A cross-sectional study on 160 patients demonstrates that caries experience is significantly associated with higher oral *Candida* carriage (Al-Amad et al., 2021). Consistent with clinical studies, *C. albicans* and *S. mutans* co-infection synergistically aggravates the onset of different types of dental caries with severe lesions, including smooth surface, and pit and fissure caries (Falsetta et al., 2014; Koo and Bowen, 2014; Thomas et al., 2016; Khoury et al., 2020; Kim et al., 2020; Du et al., 2021a). *C. albicans* inoculation significantly enhances the maturity of multi-species biofilm and sustains an acidic environment in the mixed biofilm with oral streptococci (Kim et al., 2020; Du et al., 2021a). More importantly, the growth of cariogenic bacteria *S. mutans* within the mixed biofilm under cariogenic conditions is dramatically improved by *C. albicans* (Falsetta et al., 2014; Koo and Bowen, 2014; Thomas et al., 2016; Khoury et al., 2020; Kim et al., 2020; Du et al., 2021a); Oral biofilms with *C. albicans* also express elevated levels of genes associated with acid production (*ldh*) and aciduricity (*fabM* and *atpD*) of cariogenic bacteria (Falsetta et al., 2014; Du et al., 2021a), whereas genes associated with ammonia production by commensal streptococci (*arcA* and *ureC*) are downregulated (Du et al., 2021a). In addition, the presence of *C. albicans* upregulates genes encoding CiaRH, such as *ciaR* and *ciaH*, which are implicated in the biofilm formation, acid-tolerance, and sucrose-dependent adherence of *S. mutans* (He et al., 2017). *C. albicans* significantly increases both *S. mutans* and saliva-derived biofilm demineralization ability on tooth hard tissue in an *in vitro* artificial caries model, manifested by deeper demineralized lesion and increased mineral loss (Sampaio et al., 2019; Du et al., 2021a). These promotive effects are largely accredited to *C. albicans PHR2*, and deletion of *PHR2* partially restored the microbial ecology of the polymicrobial biofilm, resulting in a biofilm with decreased acidogenicity, compromised demineralizing capability and reduced cariogenicity (Du et al., 2021a). Above all, root caries and early childhood caries (ECC) are most well-documented as associated with *C. albicans*, both commonly affected on the smooth surface of the teeth.

Root caries develops on the root surface where gingival recede and the root are exposed to the oral environment. *C. albicans* has been isolated and identified from root caries lesions for decades (Beighton and Lynch, 1993; Shen et al., 2002; Zaremba et al., 2006). Notably, longitudinal studies have revealed positive correlations between root caries increment of older adults and the presence of *Candida* in saliva (Scheinin et al., 1992; Scheinin et al., 1994). Since the development of root caries involves both hydroxyapatite demineralization and organic materials (such as type I collagen) denaturation and degradation, the increased colonization of *C. albicans* promoted by *S. mutans* may play a significant and supplementary role in the disease process. *In vitro* and animal experiments suggest that *C. albicans* hyphae can enter dentin tubules and destroy the collagen fibers with proteolytic enzyme, and this collagen hydrolase is most active in acidic environments (Nikawa et al., 1998; Klinke and Klimm, 2002; Klinke et al., 2011). In total, two characteristic colonization patterns are observed in root caries biofilm *in vivo*. In one pattern, *Candida* hyphae form a network structure extending the entire biofilm, embedded with coccoid, rod-like, and filamentous bacteria. In the other pattern, *Candida* and streptococci (usually MGS) form corncob configurations in the surface layers of the biofilm, whereas *S. mutans* clusters in microcolonies mix with other oral commensal bacteria and does not typically appear close to *C. albicans* cells (Dige and Nyvad, 2019). Our previous study revealed that *C. albicans* is detected more frequently at root carious lesions than at sound root surfaces of the same patients as well as non-caries controls. Further quantitative analysis has shown that *C. albicans* colonization follows the same trend. Meanwhile, root caries lesions possess a larger amount of *S. mutans* and reduced carriage of commensal organisms (e.g., *S. sanguinis*). Furthermore, a significant correlation between *S. mutans*/*S. sanguinis* ratio and *C. albicans* carriage is observed in all recruited subjects (Du et al., 2021a). RNA-Seq analysis has demonstrated that *C. albicans* upregulates the genes associated with *C. albicans* metabolism, sugar transportation, invasion, stress tolerance, and pH regulation in supragingival plaque of root caries, compared with this fungus in dental plaque of sound root surfaces (Ev et al., 2020). Besides *S. mutans*, another key root caries pathogen, *Actinomyces viscosus*, is often found alongside *C. albicans* in supragingival plaques of root caries patients (Shen et al., 2002). *C. albicans* co-cultured with *A. viscosus* exhibits elevated biomass of both microorganisms, and the biofilm is more acidogenic and possesses more microcolonies, which causes more damage to hydroxyapatite, *in vitro* (Deng et al., 2019).

ECC is one of the aggressive forms of dental caries that occurs in children under six-year age. Oral *C. albicans* prevalence and carrier rate are both positively correlated with the severity of ECC (Raja et al., 2010; Wu et al., 2015; Xiao et al., 2016; Lozano Moraga et al., 2017; Xiao et al., 2018b; Fakhruddin et al., 2021). Epidemiological studies have found that oral infection rate of *C. albicans* in children with ECC is higher than that in caries-free children even by different detection methods (24∼100% vs. 10∼100% in saliva, 44∼80% vs. 7∼19% in plaque, 14.7∼44% vs. 6∼7% in swab samples). More importantly, the detection rate of this fungus in carious lesions is up to 60∼100% (Xiao et al., 2018b). Absolute quantitative data show that the colonization amounts of *C. albicans* in both saliva and dental plaque collected from children with severe ECC are also significantly higher than that in the same samples of caries-free subjects (Zaremba et al., 2006; Thomas et al., 2016; Xiao et al., 2016). Furthermore, higher *C. albicans* count is detected in dental plaque of ECC children accompanied by increased *S. mutans* colonization, which is associated with higher prevalence of active caries lesion (Sridhar et al., 2020), severity (DMFT/S) of ECC (Xiao et al., 2016), and caries recurrence (Hajishengallis et al., 2017). For recurrence subjects infected with *S. mutans* strains harboring collagen-binding proteins (Cbps), higher amounts of *Candida* and *S. mutans* are identified in caries dentin compared with those infected with *Cbp*^–^ strains (Garcia et al., 2021). A recent study using 16s rRNA amplicon sequencing has revealed that oral *C. albicans* infection is accompanied by characteristic microbial communities comprising bacteria characterized by high acidogenicity and acid tolerance in severe ECC. Dental plaque of severe ECC subjects is harbored by an increased streptococci (particularly *S. mutans*), *Veillonella* and *Prevotella*, certain *Lactobacillus*/*Scardovia* species, and a decreased level of *Actinomyces* (Xiao et al., 2018a). Significant alteration in salivary fungal communities in severe ECC/ECC children is observed compared with the caries-free controls, and the fungal community is distinguished into five types based on the different oral health status, which significantly affect the bacterial profile (Tu et al., 2022).

### Oral candidosis

Oral candidosis is a common fungal disease of oral mucosa with various subtypes. There are four subtypes of oral candidosis according to the classification of Lehner (1966): pseudomembranous candidosis, acute erythematous candidosis, chronic erythematous candidosis, and chronic hyperplastic candidosis. Oral candidosis is common in “the young, the old and the sick”, also known as “a disease of the diseased”. Overgrowth of *C. albicans* on the mucosal surface is traditionally regarded as the most common cause of oral candidosis. Recently, it has been increasingly defined as a combination of fungal and bacterial biofilm-induced disease (Dongari-Bagtzoglou et al., 2009; Nett et al., 2010; Johnson et al., 2012; Xu et al., 2014b). Commensal bacteria increase not only the colonization of *C. albicans* in mucosal niches but also the persistence of *C. albicans*. The interaction between this fungus and oral bacteria may further modulate the virulence of *Candida* biofilm. Denture stomatitis is one of the chronic erythematous candidosis. Since *C. albicans* has a strong affinity for denture materials, *Candida*-associated stomatitis affects up to 60% of denture wearers (Figueiral et al., 2007; Geerts et al., 2008). *Candida* species co-exist frequently with *S. mutans* and *Staphylococcus aureus* on denture surfaces and oral mucosa of denture users (Baena-Monroy et al., 2005). The amount of *S. mutans* in saliva of active denture wearers is significantly higher than that of natural oral teeth subjects and fixed denture wearers (Beighton et al., 1990; Tanaka et al., 2009; Valentini et al., 2013). Consistent with clinical findings, *in vitro* studies exhibit that *S. mutans* and *S. oralis* aid *C. albicans* biofilm formation on hydroxyapatite, polymethyl methacrylate, and soft denture liner disks (Pereira-Cenci et al., 2008). In total, two rodent models of denture stomatitis have identified the biofilm formed on denture surfaces comprising commensal bacteria and *C. albicans* (Nett et al., 2010; Johnson et al., 2012). Inoculating *C. albicans* with MGS reveals increased colonization and biofilm efficiency in *in vitro* human oral mucosae models with salivary flow. Due to low immunity, patients with autosomal-dominant hyper IgE syndrome are predisposed to *C. albicans* infection, which has shown to maintain severe dysbiosis oral mucosal microbial communities, dominated by *C. albicans* and particularly increased abundance of *S. mutans* and *S. oralis* in patients with active infection (Abusleme et al., 2018). Compared to mono-species, co-cultured *C. albicans* with either *S. oralis* or *S. sanguinis* shows dramatically increased colonization of both *Streptococcus* and *Candida* (Diaz et al., 2012).

Besides the enrichment of colonization, interaction with oral bacteria also increases the virulence of *C. albicans*. Multi-species infections of *C. albicans* and oral bacteria are characterized by higher proportion of *C. albicans* hyphae and worse tissue invasion (Nair et al., 2001; Bamford et al., 2009; Diaz et al., 2012; Xu et al., 2014a,b; Bertolini et al., 2015; Cavalcanti et al., 2015; Xu et al., 2016; Xu et al., 2017). As a result of co-infection with *S. oralis*, *C. albicans* is better able to invade the mucosa and produces a heightened inflammatory response in comparison with infection by either microorganism alone (Diaz et al., 2012; Xu et al., 2016), reflected by the denser biofilm with longer and higher proportion of *C. albicans* hyphae extending into the submucosal compartment (Xu et al., 2016; Morse et al., 2019). *S. gordonii* promotes *C. albicans* hyphal development, which reaches 60% when *S. gordonii* is deposited first (Bamford et al., 2009). Furthermore, *C. albicans* and *S. oralis* co-infection synergistically increases the level of μ-calpain, a proteolytic enzyme capable of destroying the epithelial E-cadherin (Xu et al., 2016). *IL-18* gene expression is upregulated in reconstituted human oral epithelium infected by mixed-species biofilms, along with greater lactate dehydrogenase activity (Cavalcanti et al., 2015). The invasive ability of *C. albicans* co-infected with either *S. oralis* or *S. gordonii* is also tested in an *in vivo* oral thrush mouse model. Subjects co-infected with *Streptococcus* and this fungus exhibit significantly worse severity of tongue thrush lesions. Interestingly, quantitative analysis data of *C. albicans* from the tongue reveals that the *Candida* burdens are not significantly different with/without *S. oralis* infection (Diaz et al., 2012). Co-infection with oral *Streptococcus* and *C. albicans* leads to a stronger pro-inflammatory response compared with either single microorganism infection. Based on the microarray analysis of the mouse tongue whole genome, dual-species infected animals shows significantly upregulated genes involved in the primary categories of inflammation and neutrophilic response/chemotaxis (Xu et al., 2014b).

### Pulp and periapical inflammation

Multiple clinical data confirm that *C. albicans* is the most frequently detected fungus in infected tooth root canals (Baumgartner et al., 2000; Ashraf et al., 2007; Siqueira and Rocas, 2009; Narayanan and Vaishnavi, 2010; Kumar et al., 2015; Sakko et al., 2016; Persoon et al., 2017; Mergoni et al., 2018; Prada et al., 2019; Alberti et al., 2021). *Enterococcus faecalis* is a well-recognized pathogen of endodontic infection and post-treatment endodontic disease (Siqueira and Rôças, 2014; Delboni et al., 2017; Prada et al., 2019). The co-existence of *C. albicans* and *E. faecalis* in the oral cavity is becoming increasingly evident (Dahlen et al., 2012; Abusrewil et al., 2020). *E. faecalis* is the most common bacteria co-detected with *Candida* in oral samples (Hermann et al., 1999; Peciuliene et al., 2001). By scanning electron microscopy, it has been demonstrated that *E. faecalis* adheres to yeast and hyphal cells of *C. albicans* in infected tooth root canals as well as in dentinal tubules (Siqueira and Rocas, 2009). In an *in vitro* study, co-culturing *C. albicans* and *E. faecalis* yields a thicker and denser biofilm compared with mono-species biofilm, which exhibits higher tolerance to detrimental stresses, including alkalinity starvation, mechanical shear force, and fungicide/bactericide (Gao et al., 2016; Diogo et al., 2017; Du et al., 2021b), such as sodium hypochlorite (NaClO), ethylenediamine tetraacetic acid (EDTA), and chlorhexidine gluconate (CHX) (Diogo et al., 2017; Du et al., 2021b). The same results are also seen for antimicrobial photodynamic therapy (Diogo et al., 2017) and some new root canal medicaments (chitosan, silver nanoparticles, and ozonated olive oil) (Elshinawy et al., 2018). Consistently, significantly increased extent of periapical alveolar bone damage area is observed in an *in vivo E. faecalis* and *C. albicans* co-infection rat model, compared with mono-species infection, in conjunction with the increase in proportion of osteoclasts and decrease in osteoblasts (Du et al., 2021b). Moreover, inflammatory cytokines (TNF-a and IL-6) in periapical lesions are also upregulated by *E. faecalis*/*C. albicans* co-infection (Du et al., 2021b).

### Periodontitis and implant-related infections

*C. albicans* is also the most prevalent fungi in the periodontal pockets of periodontitis patients (Reynaud et al., 2001; Urzua et al., 2008; Al Mubarak et al., 2013; Canabarro et al., 2013; De-La-Torre et al., 2018). Clinical study has shown a higher colonization rate of *C. albicans* in patients with severe chronic periodontitis, especially *C. albicans* (De-La-Torre et al., 2018). Canabarro et al. (2013) explores that *C. albicans* is the only fungal species present in all yeast-positive chronic periodontitis cases, and *C. albicans* subgingival dental plaque colonization is related to the severity of chronic periodontitis. Co-infection with *C. albicans* and *P. gingivalis* has also been confirmed to be significantly associated with deep periodontal pockets and bleeding, contributing to active periodontitis (Oka et al., 2022). *In vitro* studies further demonstrate that *C. albicans* and *P. gingivalis* dual-species biofilm exacerbates periodontal disease, with increasing epithelial cells invasion by *P. gingivalis*. *C. albicans* may serve as a scaffold to allow *P. gingivalis* sufficient time for invasion (Tamai et al., 2011). Sequential infection initiated by *C. albicans* demonstrates a milder inflammation induced by *C. albicans* and *P. gingivalis* co-infection. *P. gingivalis* count is higher over a longer period of time in mice co-infected with *C. albicans*, suggesting that dual-species infections have a specific chronic nature (Bartnicka et al., 2020).

In terms of implant-related infections, multiple microbial biofilms on implant surfaces are thought to be the primary reason for peri-implant inflammation and peri-implant mucositis (Salvi et al., 2017; Schincaglia et al., 2017). *C. albicans* can stimulate almost all MGS species to adhere to and form biofilms on titanium surfaces. In an *in vitro* titanium-mucosal interface model, although co-cultured with *Streptococcus* does not influence pro-inflammatory cytokine responses, mucosal tissue exhibits worse damage (Souza et al., 2020). In addition, *C. albicans*/*S. gordonii* dual-species biofilms exhibit high levels of resistance to combined antifungal–antibacterial therapy (Montelongo-Jauregui et al., 2018).

### Oral cancer

Oral cancer is one of the most prevalent cancers, with most mouth neoplasms identified as oral squamous cell carcinoma (OSCC). Risk factors for oral cancer include tobacco use, heavy alcohol consumption, and human papillomavirus infection. Recently, dysbiosis in the oral microbiota has been proposed as involved in etiopathogenesis and processes of OSCC. Changes in relative abundance of specific bacteria (e.g., *P. gingivalis*, *Fusobacterium nucleatum*, and *Streptococcus* sp.) and fungi (especially *Candida* sp.) are associated with OSCC (Vyhnalova et al., 2021). While it is clear that oncological treatments can lead to changes in oral microorganisms (Sami et al., 2020), more specific mechanistic studies are needed to clarify the causality between cancer and alteration in oral microbiota composition. To our current knowledge, there is limited strong evidence on the role of fungi–bacterial interactions on OSCC development (Vyhnalova et al., 2021). Some researchers hold that microbial infections may contribute to the pathogenesis, by increasing pro-inflammatory cytokines due to microbial infection of oral mucosa (Pushalkar et al., 2011; Arzmi et al., 2019b). *C. albicans* is considered one of the major microbes contributing to oral cancer development (Kazmierczak-Siedlecka et al., 2020). Polymicrobial interactions have been shown to affect biofilm formation of *C. albicans*, *Actinomyces naeslundii*, and *S. mutans*, and biofilm effluents modulate cancer cell phenotype by increasing the adhesion of oral squamous cell carcinoma cells to extracellular matrix and enhance the expression of pro-inflammatory cytokines, particularly IL-6 and IL-8 (Arzmi et al., 2019a). This potentially cancer-promoting effect of oral microbial biofilms occurs at either the early stage of oral carcinogenesis or perhaps as an enhancement during the later stages of tumor progression. Meanwhile, metabolites from polymicrobial biofilm consisting of *C. albicans* and *S. aureus* promote the changes in proto-oncogenes and cell cycle gene expression in normal and neoplastic oral epithelial cell lines, such as *Bcl-2* and *CDKN1A* (Amaya Arbelaez et al., 2021).

## Antimicrobial resistance

Cross-kingdom interactions between *C. albicans* and oral bacteria are not only widely associated with the pathogenesis of oral disease, but will also likely change the treatment strategies for biofilm-related disease. Recent drug susceptibility studies reveal that the co-presence of *C. albicans* and oral bacteria in biofilms influences the susceptibility of either to antimicrobial agents. Eradicating *Candida*–bacterial polymicrobial biofilm-induced diseases is challenging (Montelongo-Jauregui et al., 2016; Diogo et al., 2017; Elshinawy et al., 2018; Kim et al., 2018; Chinnici et al., 2019; Ikono et al., 2019; Du et al., 2021b; Gong et al., 2021; Kulshrestha and Gupta, 2022). Montelongo-Jauregui et al. (2016) reports that in both monotherapy and combination therapy with commonly used antifungals and antibacterial antibiotics, *C. albicans*/*S. gordonii* mixed-species biofilm becomes more resistant to antimicrobial treatments at all doses regardless of whether they are cultured on conventional media or synthetic saliva. Ampicillin resistant polymicrobial biofilms consisting of *S. gordonii* and *C. albicans* appear to be controlled by transcription factors from *C. albicans* (Sfl2, Tec1) (Chinnici et al., 2019).

Polysaccharides secreted by microorganisms in biofilm may play an important role in antibiotic resistance, which might prevent drug penetration and provide protection for the microorganisms. *In situ* generated EPS by *S. mutants* directly binds and sequesters fluconazole, reducing drug uptake and intracellular transport, and the combination of topical bactericidal povidone iodine with fluconazole increase can completely suppress *C. albicans* carriage and mixed-biofilm formation without increasing bacterial killing activity *in vivo* (Kim et al., 2018). Meanwhile, *C. albicans* and its secreted cell wall polysaccharide material, especially β-1,3-glucan cell wall component, significantly enhance the tolerance of *S. aureus* to drugs. Fluorescence confocal time-lapse microscopy reveals the impairment of drug diffusion through the mixed biofilm matrix. By inhibiting the production of the fungal polysaccharides, a specific antifungal agent indirectly sensitized the bacteria to antimicrobials (Kong et al., 2016).

## Conclusion and perspectives

The interactions between *C. albicans* and oral bacteria play an important role in oral microecology and are closely associated with the occurrence, development, and treatment of biofilm-related oral diseases. Understanding such symbiotic interactions with clinical relevance between microbial species in biofilms will greatly aid in disease prevention and overcoming the limitations of current therapies. Studies focusing on the mutualistic interactions between *C. albicans* and specific pathogens *in vitro* are fundamental in simplifying the phenomenon and exploring the underlying mechanisms, making great progress for reveal the underlying mechanisms. Additionally, it is necessary to recognize the fact that *Candida*–bacterial interaction is not only driven by microbial communication but also relies on the influence of environmental conditions, microbial communities, and host factors. The limitation of most *in vitro* study not being able to reproduce the oral environment is still a problem that requires a breakthrough. *In silico* or computational studies performed with genomic and metabolic pathway comparisons may help to capture important interaction mechanisms or molecule in complex interaction networks between *C. albicans* and oral bacteria. Further in-depth studies using models mimicking health and disease situations and clinical trials are still required to delineate the underlying molecular mechanisms. As the threat of antimicrobial resistance increases, the need for new antimicrobial and antifungal agents is reaching a tipping point. Identifying effective therapeutic techniques for *Candida*–bacterial polymicrobial biofilm is a new approach, rather than focusing only on the specific pathogen (Hwang, 2022). Although there is a growing awareness of the importance of combatting cross-kingdom biofilms, the majority of treatments rely on broad-spectrum antimicrobial activity that can kill both fungus and bacterium or supplemented with antifungal drugs. New therapeutics targeting the binding mechanism between *C. albicans* and streptococci are currently being investigated. For instance, Kim et al. (2021) presents that mannan-degrading exo- and endo-enzymes target GtfB–mannan interactions in this cross-kingdom consortium and are highly effective in reducing biofilm biomass without killing microorganisms, as well as alleviating the production of an acidic pH environment conducive to tooth decay. Furthermore, recombinantly expressed human and mouse serum amyloid A1 (rhSAA1) proteins promote cell aggregation and target the *C. albicans* cell wall adhesin Als3 (Gong et al., 2020). Computational methodologies have become crucial components of many programs used in pharmaceutical production for discovery of new antibacterial targets that may decrease virulence of cariogenic microorganisms present in dental biofilms and quickly predict their spectrum and selectivity (da Silva et al., 2014; Rivera-Quiroga et al., 2020; Alharbi et al., 2022). Indeed, we still have a long way to go in finding effective treatments. It would be meaningful to combine *in silico*, *in vitro*, and *in vivo* studies, examining bacterial–fungal interactions in a high-throughput manner to systemically evaluate both positive and negative effects on proliferation and virulence. Further researches focus on molecular targets and signaling pathways of *C. albicans*, and oral commensal bacteria interactions and the effects on the virulence of fungi–bacteria cross-kingdom biofilms are necessary.

## Author contributions

QD: conceptualization, methodology, software, investigation, and writing—original draft preparation. BR: writing—review and editing. XZ: resources and validation. LZ: supervision, project administration, and writing—review and editing. XX: writing—review and editing, visualization, and funding acquisition. All authors have read and agreed to the published version of the manuscript.
